# The Therapeutic Value of Some Medicines in the Treatment of Hemorrhagic Conditions of the Uterus

**Published:** 1887-12

**Authors:** C. D. Palmer

**Affiliations:** Cincinnati; Professor of Obstetrics and Gynecology, Medical College of Ohio; Obstetrician to the Cincinnati Hospital


### *THE THERAPEUTIC VALUE OF SOME MEDICINES IN THE TREATMENT OF HEMORRHAGIC CONDITIONS OF THE UTERUS.*

By C. D. PALMER, M. D., Cincinnati.

Professor of Obstetrics and Gynecology, Medical College of Ohio; Obstetrician to the Cincinnati Hospital.

The author, in a paper read before the American Gynecological Society, said, in the abstract, as follows:

Among the medicines which time and experience have proven to be the most useful in the treatment of menorrhagia and metrorrhagia are ergot, digitalis, canabis indica, bromide of potassium, arsenic and gallic acid. No one of these is more frequently prescribed, and with better success, and is better understood, than ergot. It is curative in some cases, benefits many, and may aggravate a few. It is singularly adapted to the relaxed muscular fibres, dilated and engorged blood-vessels. Chronic hyperæmia, of an active or passive kind, chronic metritis, in its first stages, and sub-involution, which are attended by increased menstrual flux, or hemorrhage, are controlled by ergot. The more soft, flabby and succulent the uterus is with blood, the better the ergot will act. The treatment of uterine fibroids, between the well-earned claims of galvanism and the fully-tested powers of ergot, the field of laparo-hysterotomy is destined to be greatly and very properly limited.

Digitalis, in proper cases, in good form, the infusion, is a remarkably reliable agent in cases properly selected. It is temporarily beneficial in uterine hemorrhages, resultant on cardiac disease, mitral insufficiency, and may result in a cure in other cases. An atonic condition of the circulation, a weak heart, with slow or rapid action, low arterial tension, may be combated by digitalis instead of ergot.

Cannabis indica is a more uncertain drug. It varies much in its strength and in its action, in different persons. It stimulates the cerebro-spinal and sexual centers, exciting uterine contractions. Its mode of action is not well understood, its indications are imperfectly defined, and its effects uncertain and unreliable.

Bromide of potassium, for certain menorrhagic states, is a remedy of no mean power. It acts as a sedative to the sexual apparatus, especially the ovarian functions, and, theoretically, we would expect it to be most efficacious in conditions of sexual excitement, ovarian irritation and congestion. Clinically, the theory has been found to be a fact. For the menorrhagia of ovarian, rather than uterine, origin, it is to be chosen. For the hemorrhage of sub-acute and chronic pelvic peritonitis, it is our best remedy. It is very possible that the bromide is more certain in its effect in arresting menstrual than non-menstrual hemorrhage.

Arsenic, on the other hand, has its influence directed more particularly to the uterus. Not prompt in action as ergot, digitalis and the bromides, yet, in selected cases, it is none the less decided. Arsenic, in medicinal doses, acts as a tonic and stimulant, improving bodily nutrition, with a special direction to the skin.

It is now considered to have a very similar effect on the mucous membrane to that on the skin, the morbid conditions of which are much the same.

Chronic endometritis, of long standing, with or without fungoid degeneration, expressing itself by a stubborn leucorrhœa and metrorrhagia, the causative relation of which is seemingly constitutional, rather than local, is often materially improved by long courses of arsenic; in the lymphatic temperament, and in constitutions of the blonde type, arsenic is apparently most inefficacious.

My general experience enables me to speak very highly of arsenic in two forms of menorrhagia: that of growing girls and young women, nullipara chiefly, in whom menstruation is not necessarily too free, but appears too frequently and continues too long; a vicious habit of irregularity of menstrual function, from some cause, becomes established, highly detrimental to the health. Small doses of Fowler's solution, gtt. III., continued during the interval, as well as the menstrual period, has rarely failed, in his hands, to correct the irregularity. The other class is the menorrhagia of the climacteric, either as to the time, quantity or duration. In this, it is not so successful, as, unfortunately, this is often due to some malignant disease. Arsenic is a good remedy in menorrhagia of malarial origin, as also in chorea, associated with menorrhagia in growing girls.

Gallic acid is the best of the astringent class, and is quite efficacious in restraining hemorrhage, resultant on uterine atony. Its disagreeable taste, and bad effect on the digestive functions, must limit its use.

Of the power of active cathartics, especially the so-called chologogues—mercury in particular—in promptly checking, at times, uterine hemorrhage, all observing practitioners must attest. Such medication is indicated in menorrhagia of hepatic or splenic origin. Unload the portal circulation, and we relieve the pelvic engorgement.

Iron, as a rule, promotes pelvic congestion, and its use, in these cases, is almost invariably injurious. An increased and perpetuation of a normal and abnormal uterine flux, as a result of a hydræmic state of the blood, lacking power of spontaneous hæmostasis, offers a condition in which the muriated tincture of iron is highly beneficial.

Viburnum prunifolium is recommended in systemic cases, and dysmenorrhœa associated with menorrhagia.

Hydrastis canadensis is reported upon very favorably by Schatz, of Rostock, and Slaviansky, of St. Petersburgh. It is considered, by these authorities, to be a vaso-constrictor of congested states of congested mucous membranes. In menorrhagia, from whatever cause, in hemorrhages, due to metritis, endometritis, myomas, incomplete involution, they have found it invaluable.

Gossypium herbaceum, after extensive trials in all forms of uterine hæmorrhage by Dr. Henry Garriqes, has been found quite efficacious. He relieved 125 out of 139 patients. He gives it in a decoction, and has found it preferable to all other remedies in a majority of cases, the effects being most noticeable in neoplasm.

Hamemelis virginica is a drug which is entitled to a higher position among our therapeutic remedies than it has occupied. I think it superior to viburnum, hydrastis, gossypium, and, in some cases, to ergot. It does not seem equally potent for all kinds and conditions of uterine hemorrhage. For the sudden outburst of a flow, for active, profuse hemorrhage, it is inferior to ergot. For a slow and long-continued hemorrhage, when the blood is dark and venous, and the hemorrhage is passive in character, a symptomology indicative of passive engorgement and relaxation, it is a remedy par excellence. These are conditions ever present in flabby, enlarged, sub-involuted uteri, after delivery and abortion, also in some forms of chronic endometritis, before or after the removal of fungosities, and in chronic retro-version, some fibroids, etc. I have been pleased with the effect of hamemelis on hemorrhage in several cases of papilloma of the bladder. The so-called Pond's Extract, a preparation of hamemelis, and a colorless, distilled product, are unworthy of confidence. The best preparation is the officinal fluid extract, in doses of a few drops up to two drachms. Small doses, often repeated, are recommended, but large ones should be used when decided effects are urgent. Hamemelis is somewhat tonic and sedative, slightly astringent and decidedly hæmostatic. The virtues of the drug cannot reside alone in the tannic and tallic acids which it contains, for equal quantities of the astringent fail to produce similar results. Hamemeline has been extracted from it, but whether this represents the chief active ingredient, is uncertain.

In endeavoring thus imperfectly to outline the especial fields of usefulness of these medicinal remedies, which may be depended upon in uterine hemorrhages, nothing should be so construed that a single one of the number ought to supersede local, mechanical and surgical interference when indicated. Occasionally, general medication alone will suffice more often; it goes hand in hand with other management.

